# Tuberculosis infection following immune checkpoint inhibitor treatment for advanced cancer: a case report and literature review

**DOI:** 10.3389/fimmu.2023.1162190

**Published:** 2023-05-25

**Authors:** Chen Lin, Guixiang Xu, Shuyan Gao, Tao Feng, Shuang Li

**Affiliations:** Department of Respiratory and Critical Care Medicine, Shengli Oilfield Central Hospital, Dongying, Shandong, China

**Keywords:** immune checkpoint inhibitors, tuberculosis, drug-related adverse reactions, interferon-γ release assay, tuberculosis screening

## Abstract

**Objective:**

To investigate the clinical features of active tuberculosis (TB) infection due to immune checkpoint inhibitors (ICIs) treatment in patients with advanced cancer.

**Methods:**

We report the diagnosis and treatment of a case of pulmonary malignancy (squamous cell carcinoma, cT4N3M0 IIIC), secondary to active TB infection following ICIs therapy. Moreover, we summarize and analyze other related cases collected from the China National Knowledge Infrastructure (CNKI), Wanfang Database, PubMed, the Web of Science, and EMBASE (up to October 2021).

**Results:**

A total of 23 patients, including 20 males and 3 females who were aged 49-87 years with a median age of 65 years, were included in the study. Twenty-two patients were diagnosed by Mycobacterium tuberculosis culture or DNA polymerase chain reaction (PCR), while the remaining patient was diagnosed by tuberculin purified protein derivative and pleural biopsy. One case had an interferon-gamma release assay (IGRA) to rule out latent TB infection prior to the application of ICI. Fifteen patients received an anti-tuberculosis regimen. Among the 20 patients with a description of clinical regression, 13 improved and 7 died. Seven of the patients who improved were treated with ICI again and four of them did not experience a recurrence or worsening of TB. The case diagnosed in our hospital also improved after receiving anti-TB treatment after stopping ICI therapy, and continued chemotherapy on the basis of anti-TB treatment, and his condition is relatively stable at present.

**Conclusion:**

Due to the lack of specificity of TB infection following ICIs therapy, patients should be followed for fever and respiratory symptoms for 6.3 months after drug administration. It is recommended that IGRA should be performed before ICIs therapy and the development of TB during immunotherapy in patients who are positive in IGRA should be closely monitored. The symptoms of TB in most patients can be improved with ICIs withdrawal and anti-TB treatment, but there is still a need to be alert to the potentially fatal risk of TB.

## 1 Introduction

Mycobacterium tuberculosis (MTB) poses a huge burden on the whole world, with 1.7 billion people worldwide estimated to be potentially infected with tuberculosis (TB) (1). Several studies have shown that patients with hemopathy and malignant solid tumors like head and neck cancers and lung cancers are at increased risk of developing TB (2, 3). Some guidelines, including that of the United States Preventive Services Task Force (USPSTF), suggest patients at higher risk of developing active TB should take latent TB screening (4).

Immunotherapy represents an important and growing area of clinical oncology. Immune checkpoint inhibitors (ICIs) exert their antitumor activity by inhibiting the suppression pathway of immune cells, which can enhance the activity of immune cells and reduce T cell depletion (5). Currently, approved checkpoint inhibitors include cytotoxic T lymphocyte-associated antigen-4 (CTLA4) inhibitors, programmed death receptor-1 (PD-1), and programmed death ligand-1 (PD-L1) inhibitors. Despite the significant effects of ICIs, immune-related adverse events usually occur during treatment, with increasing reports of MTB infections occurring during the treatment of ICIs (**Table S1**). Clinical studies have shown that immune checkpoint pathways, such as the PD-1 and PD-L1 axes, play an important role in immune homeostasis in TB, and that the deficiency of PD-1 leads to the worsening of TB in animal models (6). After being exposed to tuberculosis, PD-1-deficient mice developed necrotizing pulmonary lesions with abundant acid-fast bacilli, characterized by many granulomas, local necrosis, and neutrophil infiltration (7). Laboratory tests showed a greatly increased MTB-specific CD4 T cell immune response and high levels of inflammatory cytokines in the lung of PD-1 deficient mice (7). This suggests that inhibition of PD-1 may enhance CD4 T cell-mediated immunity, leading to excessive inflammatory response and tissue destruction in the context of TB infection, thereby reactivating tuberculosis (6, 8, 9). In addition, Langan and others (10) summarized all published cases of TB infection during ICIs immunotherapy and pointed out that patients with non-small cell lung cancer treated with ICIs are at increased risk because activation of TB may result from activation of specific immune cell subsets. This evidence suggests that ICIs treatment may directly or indirectly lead to TB infection, but the exact mechanism remains unclear.

In the present study, a case of active TB associated with the application of camrelizumab in 2022 was studied and a systematic review of the collected related literature was taken to further elucidate the potential impact of ICIs on the development of active TB.

## 2 Subjects and methods

### 2.1 Subjects

One patient with active TB infection following camrelizumab treatment who was admitted to Shengli Oilfield Central Hospital and patients collected through literature search with TB associated with ICIs.

### 2.2 Methods

We first consulted and organized the electronic medical records of the patient admitted to Shengli Oilfield Central Hospital to form a case report. Moreover, we searched through the China National Knowledge Infrastructure (CNKI) and Wanfang Database with the terms ‘ICIs’ and ‘TB’ up to October 2021, but no similar reports were found in China. Then, the compound terms ‘TB’ and ‘PD-1’ or ‘PD-L1’ or ‘CTLA-4’ or ‘pembrolizumab’ or ‘nivolumab’ or ‘caliplimab’ or ‘atezolizumab’ or ‘avelumab’ or ‘duvalumab’ or ‘ipilimumab’ or ‘temlimumab’ were searched for on PubMed and the Web of Science. Furthermore, ‘TB’ and (‘pembrolizumab’, ‘nivolumab’, ‘cymplimab’, ‘atezolizumab’, ‘avelumab’, ‘duvalumab’, ‘ipilimumab’ or ‘temlimumab’) were searched for on EMBASE. Each of the obtained results was relevant to the topic of TB in ICIs therapy, and the data provided in the cases were relatively complete.

**Figure 1:**
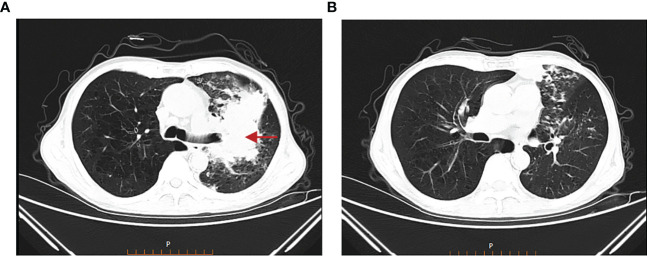
Chest CT on 05 March 2022. **(A)** Truncated bronchi in the upper lobe of the left lung, multiple small patchy and nodular high-density shadows in the surrounding lung. **(B)** Multiple small nodules and small patchy high-density shadows in the lower lobe of the left lung.

**Figures 1A, B** show the lung window scans of enhanced chest CTs, which describe the contents of the left upper lobe of the lung with bronchial truncation and inflammation of the left lower lobe of the lung, respectively. All scans were from different slices of the same patient on the same date. The following comparative CT slices were all obtained from the same machine, and each slice was corresponding to the previous one, so they can be directly compared.

In addition, an Excel data extraction sheet was applied to extract relevant data on patients from clinical records and literature, including general data (such as gender, age, region, and primary tumor), ICIs application, applications of other immunosuppressants, the development of TB (time lag from ICIs administration to TB occurrence and laboratory results of TB), treatment and regression of TB, and the reapplication of ICIs. Finally, we performed statistical analysis of the collected data.

## 3 Results

### 3.1 Case report

The patient, a 68-year-old male, was admitted to our hospital because of “cough and sputum with breathlessness for 3 months”. The patient presented with cough and sputum 3 months ago. Specifically, the patient had a paroxysmal cough with white mucous sputum, accompanied by chest distress, shortness of breath, and numbness of both lower limbs. He had a previous history of smoking for 50 years (20 cigarettes/day) but had quit smoking for 5 months. The rest of his past medical history was negative.

Physical examination: general condition was acceptable, superficial lymph nodes were not palpably enlarged, and the breath sounds of both lungs were rough without rhonchus and moist rales. No abnormalities were found on cardiac or abdominal examination.

The examinations undertaken are listed in **Table 1**. All images were taken by contrast-enhanced CT.

**Table 1:** The examination results of the patient in our hospital.

Examination	Results		
	Indicators	Normal person	Patient
Chest CT plain scan + enhanced scan (**Figure 1A, B**, 05 March 2022)	Mass	–	~ 12.6 × 7.6 cm
	Truncated bronchi	–	+
	Pulmonary artery	Normal	Localized thinning
Cranial MRI plain scan + enhanced scan (06 March 2022)	Signal shadow	Normal	Abnormal
Tracheoscopy (09 March 2022)	Cauliflower-like neoplasm	–	
	Bronchial tubes of the bilateral lobes	Normal	Normal
Tumor markers (03 March 2022)	CEA	≤ 5 ng/ml	(+) 6 mg/ml
	CA125	0-35 U/ml	88.18 U/ml
	cytokeratin	0.10-4 ng/ml	12.91 ng/ml
	NSE	< 13 μg/ml	33.72 ng/ml
Blood routine	Normal		
Biochemistry analysis			
Coagulation function			
Echocardiography			
Electrocardiogram			
Whole-body bone scintigraphy			
Pathological findings	CK5/6	<10% (-)	+
	CK7	–	–
	Ki-67	–	+, (50%-60%)
	Napsin A	–	–
	P40	–	+
	TTF-1	–	–
	P63	–	+
	bacteria	Normal	
Chest CT (**Figure 3A, B**, 22 May 2022),	Cancer area	–	Less extensive
	Mass	–	More extensive
13-item nucleic acid test (lavage fluid)	MTB		+
Acid-fast bacillus stain (tracheoscopy lavage solution)	Acid-fast bacillus	–	+
CT enhanced scan (19 August 2022)	Mass	–	Less extensive

1. Chest CT plain scan and enhanced scan: (**Figures 1A, B**, 05 March 2022): a mixed-density mass measuring approximately 12.6 × 7.6 cm was found on the left upper lobe of the lung, with truncated bronchi in the upper lobe of the left lung, localized thinning of the left pulmonary artery, and multiple small patchy and nodular high-density shadows in the surrounding lung; multiple small nodules and small patchy high-density shadows were found in the lower lobe of the left lung.

2. Cranial MRI plain scan and enhanced scan (06 March 2022): abnormal lesion signal shadow was found in the anterior horn of the left lateral ventricle, which was presumed to be a cavernous hemangioma.

3. Tracheoscopy (09 March 2022): cauliflower-like neoplasm was found to block the orifice in the upper lobe of the left lung, and the opening of the lower lobe was slightly compressed and narrowed; no abnormalities were found in the bronchial tubes of the bilateral lobes.

4. Tumor markers (03 March 2022): carcinoembryonic antigen (CEA) was 6mg/ml, CA125 was 88.18U/ml, section 19 of cytokeratin was 12.91ng/ml, and neuron-specific enolase (NSE) was 33.72ng/ml.

There were no abnormalities in the routine blood test, whole-body bone imaging, biochemistry analysis, blood clotting, echocardiography, and electrocardiogram.

Pathological findings: the moderately differentiated squamous cell carcinoma was revealed (through bronchoscopy biopsy of left upper lobe lung). Immunohistochemical findings: CK5/6 (+), CK7 (-), Ki-67 (+, 50%-60%), NapsinA (-), P40 (+), TTF-1 (-), and P63 (+). No abnormalities were found in etiological examinations for bacteria, fungus, *M. tuberculosis*, Pneumocystis carinii, *Actinomyces*, nocardiosis, and cytomegalovirus.

Final diagnosis: there was a malignant tumor (squamous cell carcinoma, cT4N3M0 IIIC) with obstructive pneumonia in the upper lobe of the left lung. Since the patient had no surgical indications, he was suggested for chemotherapy combined with radiotherapy and PD-L1 testing. Genetic testing showed negative results for EGFR, ALK, ROS1, and PD-L1 expression.

**Figure 2:**
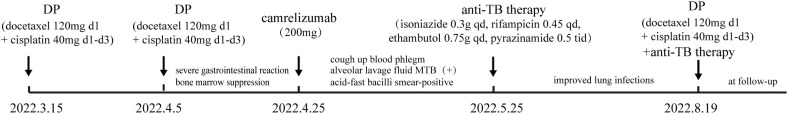
The timeline of treatment of the patient in our hospital.

**Figure 3:**
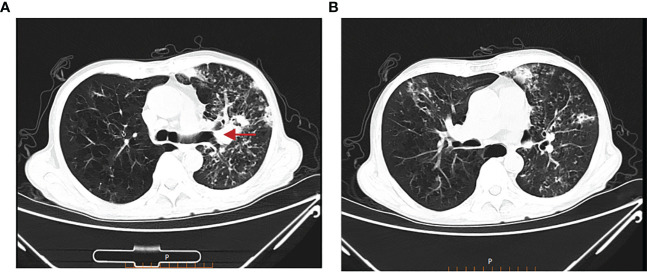
Chest CT on 22 May 2022. **(A)** The lung cancer in the upper lobe of the left lung was less extensive than before. **(B)** Multiple small nodules and small patchy high-density shadows in both lungs were more extensive than before.

**Figure 4:**
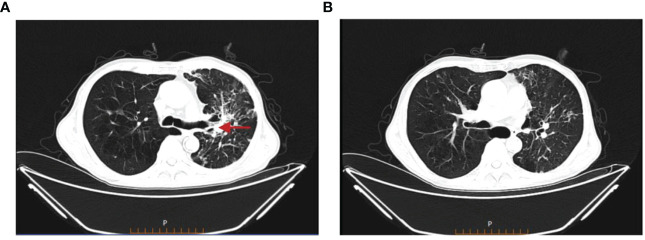
Chest CT on 19 August 2022. **(A)** The primary focus of cancer in the right lung was stable. **(B)** The nodular and small patchy high-density shadows in both lungs were less extensive than before.

Treatment:

Treatment course: a brief description of the treatment course is shown in **Figure 2**. After excluding contraindications, the first cycle of DP chemotherapy (docetaxel 120mg d1 + cisplatin 40mg d1-d3) was administered on 15 March 2022. Here, the chemotherapy went smoothly and the patient did not have any complaints. Then, the second cycle of chemotherapy was administered on 05 April 2022 with the same regimen as the first cycle. During chemotherapy, the patient had a severe gastrointestinal reaction and bone marrow suppression, with white blood cells falling to 1.3 × 10^9^/L and neutrophils dropping to 0.23 × 10^9^/L. Also, the patient was given symptomatic treatment such as leuke-raising injections but refused to accept another cycle of chemotherapy. After excluding contraindications, he was treated with 200 mg of camrelizumab for immunotherapy on 25 April 2022. On 21 May 2022, the patient began to cough up blood in phlegm with a volume of about 20 ml and he was reviewed using a chest CT on 22 May 2022 (**Figures 3A, B**), which showed that the lung cancer in the upper lobe of the left lung was less extensive than before, while multiple small nodules and small patchy high-density shadows in both lungs were more extensive than before, which could not exclude pneumonia or infection associated with ICIs. The patient was diagnosed with a tracheoscopy and a 13-item nucleic acid test for respiratory pathogens in the lavage fluid, which was positive for MTB. Moreover, the acid-fast bacillus stain test was positive. Therefore, the patient was considered to have active TB. Multidisciplinary oncology consultation suggested that the patient should stop immunotherapy and be treated with a four-drug anti-TB regimen of isoniazide, rifampicin, ethambutol, and pyrazinamide. After 3 months of anti-TB treatment, the patient was reviewed using an enhanced CT scan on 19 August 2022, which showed that the primary focus of cancer in the right lung was stable and the nodular and small patchy high-density shadows in both lungs were less extensive than before (**Figures 4A, B**). Finally, the lung was stable, and no further ICIs were administered.The patient is currently in a relatively stable condition and is being followed up.

### 3.2 Patient characteristics

There were 23 patients, 20 men and three women aged 49-87 years, with a median age of 65 years. Among the 19 patients with a specified race, there were six Caucasian, two Japanese, three Chinese, two Greeks, one Vietnamese, one Belgian, and four Korean. Of these patients, there were twelve cases of non-small cell lung cancer, one case of undifferentiated lung cancer, five cases of melanoma (including one case of ocular melanoma), two cases of oral squamous cell carcinoma, one case of nasopharyngeal carcinoma, one case of Hodgkin lymphoma, and one case of Merkel cell carcinoma.

### 3.3 Application of immunotherapy and other therapies

All patients were treated with PD-1 inhibitors. Only one patient (9) received the treatment of CTLA-4 inhibitor (Ipilimumab) before pabrolizumab. Thirteen patients were treated with nivolumab, eight patients were treated with pembrolizumab, one patient was treated with atezolizumab, and one patient was treated with camrelizumab. Thirteen of 18 patients with prior treatment data received chemotherapy. Among the 20 patients that developed active MTB infection, four patients (10, 11) required steroids or infliximab to treat immune-related adverse events (irAEs) and a further two patients (12, 13) had immune-related adverse events such as Sjogren’s syndrome and adrenal insufficiency before the diagnosis and treatment of TB, but the specific treatment of immune-related adverse events for these patients was not documented. There were no reports about the application of immunosuppressive drugs in these cases except for the six patients mentioned above who might have had immunosuppressive drugs applied to treat immune-related adverse reactions.

### 3.4 TB screening and occurrence before the treatment

Only one patient (14) screened negative for TB with an IGRA before starting with immunization. Two other patients with TB were initially thought to have developed secondary lesions of primary cancer. One patient (13) with melanoma developed a right upper lobe pulmonary nodule during treatment with pabrolizumab. One patient (11) with lung adenocarcinoma presented with pericardial thickening, which was also initially thought to be related to cancer until it had not subsided with the treatment of nivolumab.

Two of the 23 patients were presumed to have latent TB before the application of ICIs. One of them (8) was from Vietnam, where TB is endemic, and the other (15) had contact with a patient with active TB 10 years ago. For three other patients (12, 16, 17) it was explicitly stated that TB represented reactivation, but the reasons for this were not given.

### 3.5 Diagnosis of TB

Twenty-two of the 23 cases were confirmed to have TB by the culture of Mycobacterium TB or by PCR analysis of TB DNA. The analytical specimens were mainly sputum or bronchoalveolar lavage fluid, but reactivation of TB in other organs (pericardium, bone, liver, and gastrointestinal tract; one case each) was also recorded. The remaining case (18) was diagnosed mainly by purified tuberculin skin test and pathological findings of caseous granuloma on pleural biopsy and symptomatic remission with four anti-TB drugs. The imaging mainly showed new or worsening nodules, whose pathological biopsies were all granulomatous inflammation. The time lag from initiation of ICIs therapy to the diagnosis of TB of these patients ranged from 1 month to 2 years, with a median of 6.3 months.

### 3.6 Effectiveness of anti-TB and ICIs therapy

Immunotherapy was discontinued in all but five patients (10, 18, 19) (three (19) of these cases are unknown) after the diagnosis of TB. Seven patients restarted ICIs therapy, two of whom (11, 20) after finding resolution of TB symptoms and no recurrence one month after discontinuation of ICIs. Four other patients (12, 13, 21, 22) suspended immunotherapy after diagnosis of TB but restarted with immunotherapy before completion of anti-TB treatment. One patient (8) restarted with ICIs therapy at the time of progression of the primary disease, with the metastatic mass shrinking.

The specific anti-TB regimens of eight patients were not listed. Of the other 15 patients (including the patient in the study), 11 of them were initially treated with isoniazid, rifampicin, pyrazinamide, and ethambutol (8, 10, 13, 15, 19, 21, 22), one of them (16) was initially treated with rifampicin, isoniazid, and ethambutol, and two (18) of them were treated with four drugs not specified. Some patients changed their regimens due to adverse events during treatment.

Seven patients died during follow-up. The first (18) died intraoperatively while attempting to remove a tuberculoma that caused spinal cord compression. The second (8) died of intestinal perforation due to disseminated TB. The third (17) died of respiratory failure secondary to pneumonia, but it was unclear whether this was related to TB. The fourth (22) had apparently an improved TB condition and restarted with nivolumab, but eventually died of respiratory failure in the progression of lung adenocarcinoma. The fifth (10) died of acute respiratory failure after 3 days of anti-TB treatment. The sixth (19) died due to peritonitis, and the seventh (19) died of the progression of lung adenocarcinoma. Of these seven deaths, two were directly attributable to the TB, and the third might have been directly or indirectly related to the TB as he died of respiratory failure after developing pneumonia in the setting of active TB.

## 4 Discussion

Our results suggest that inhibition of the PD-1/PD-L1 pathway is associated with the development of TB, and the ICIs leading to TB was a PD-1 inhibitor in all cases. Moreover, there were no cases of TB disease found that occurred during treatment with CTLA-4 inhibitors. In the majority of cases, TB-related signs and symptoms improved with TB treatment, but two patients died of TB-related complications. Moreover, our findings also suggest that active TB can develop in a variety of organs other than the lungs.

**Figure 5:**
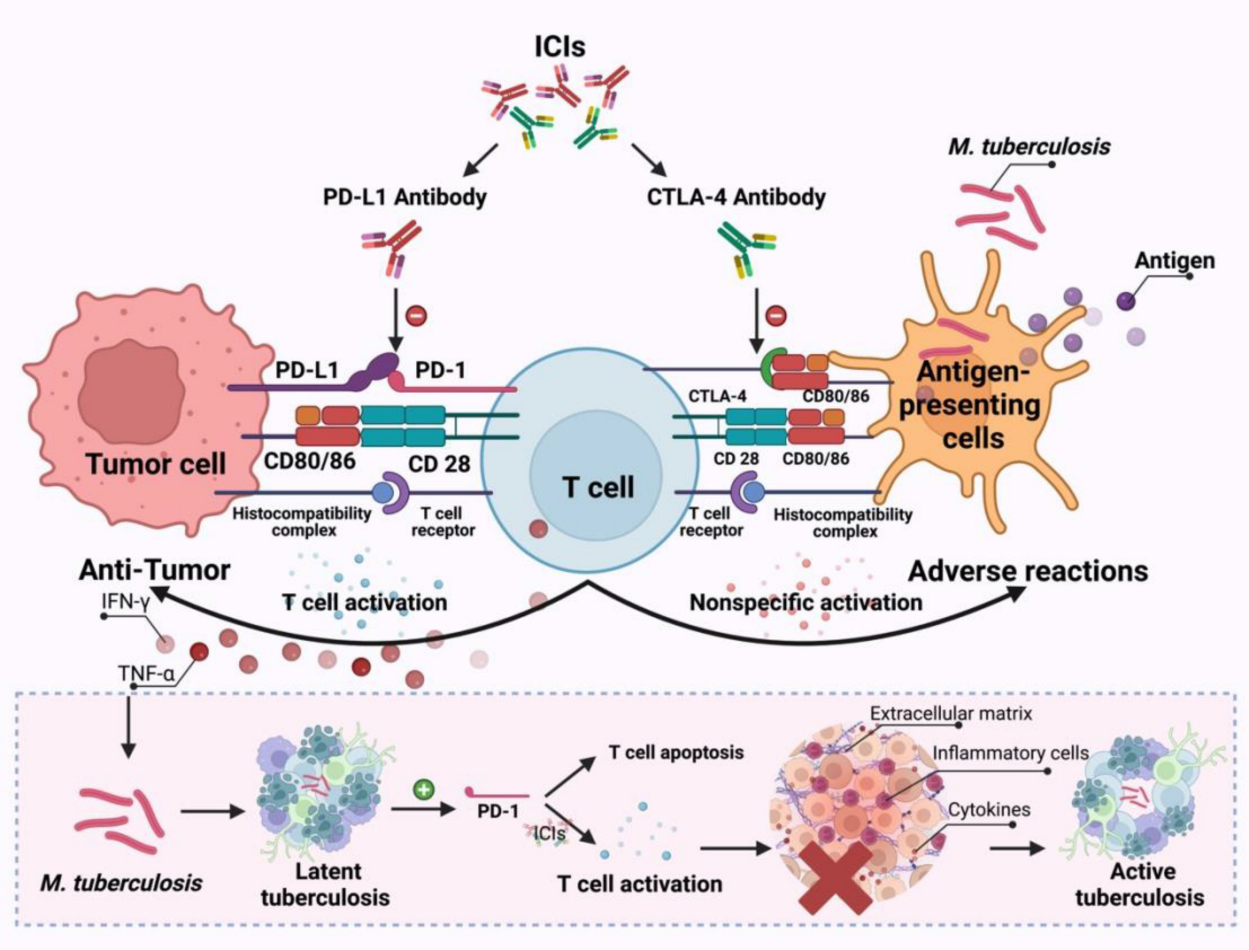
The possible mechanisms through which ICIs treatment promotes the development of TB infection.

It has been demonstrated that the PD-1/PD-L1 pathway is closely related to the pathophysiology of TB. PD-1 (CD279/PDCD1) is a cell surface inhibitory receptor that is expressed on activated T and B cells upon binding to the Ag receptor. PD-1 binds to either of its two ligands, PD-L1 (B7-H1/CD274) and PD-L2 (B7-DC/CD273), inhibiting T cell proliferation and cytokine secretion. ICIs can relieve the immunosuppression of T cell activation by tumor cells, and promote the activation and proliferation of T cells, thereby killing tumor cells. When the body is infected with TB, MTB is phagocytosed by macrophages and presented to T cells after processing by antigen-presenting cells, and then T lymphocytes secrete a variety of cytokines, such as γ interferon (IFN-γ) and tumor necrosis factor (TNF)-α, to activate the anti-TB activity of macrophages. When TB granulomas are formed, the body presents latent tuberculosis infection, at which the expression of PD-1 increases, inducing apoptosis of T cells. ICIs can block PD-1, restore the function of lymphocytes, release inflammatory cytokines such as IFN-γand TNF-αin T cells again, and excessive inflammatory cells and cytokines destroy the extracellular matrix, which is conducive to the growth of MTB, disrupts the homeostatic control of latent pulmonary tuberculosis infection, and active tuberculosis infection occurs (**Figure 5**). Studies have shown that the type I IFN signaling pathway is enhanced in patients receiving PD-1 blockade. TB activity represents an enhanced immune response to a pre-existing pathogen in the presence of PD-1 or PD-L1 inhibition, which is similar to the immune reconstitution inflammatory syndrome in HIV (human immunodeficiency virus) (14, 18). Furthermore, corticosteroids may have a role in the treatment of the immune reconstitution inflammatory syndrome as it is associated with an enhanced immune response. Therefore, the developing rate of immune reconstitution inflammatory syndrome associated with TB in HIV patients can be reduced with the combined application of prednisone and antiretroviral therapy (ART) (23). In this research, three (11, 21, 22) patients received the combined treatment of corticosteroids and anti-TB at some point after the diagnosis of TB and all of them were considered to be in remission or showed improvement. The TB of five other patients had also been relieved without the application of steroids, which shows that whether steroids play a role in the treatment of patients who develop active TB during ICIs therapy needs to be further investigated, and the potential benefits of corticosteroids must be weighed against their attenuating effect on immunotherapy in the process of application (24, 25).

TB screening in high-risk patient groups, particularly those exposed to epidemiological risk factors for TB, should be applied to patients before the initiation of immunotherapy. In this study, 10 of the 19 patients were from East or South East Asia where TB is endemic. If patients receiving anti-PD1 antibodies in areas with high TB prevalence develop fever and respiratory symptoms, the reactivation of TB should be considered after respiratory infection and pneumonia has been excluded. Therefore, latent TB screening needs to be applied before starting ICIs therapy in TB-endemic countries. A previous study has shown that adequate treatment of latent TB infection significantly reduces the risk of active TB in these patients (26). Although immunosuppression is also a risk factor for TB, the extent to which immunosuppression promotes the development of active TB in cancer patients treated with ICIs is unknown. Most of the patients in the study received chemotherapy before ICIs therapy, which may have contributed to immunosuppression. Whether the concomitant use of immunosuppressive drugs such as glucocorticoids and ICIs therapy are associated with the development of active TB disease still needs to be confirmed in larger studies. It cannot be ruled out whether the development of tuberculosis is associated with high doses of steroid drugs. In the above cases, no concomitant use of immunosuppressive drugs was reported, except for the case of four patients who required steroids or infliximab to treat irAEs and two patients who might require immunosuppressive drugs such as glucocorticoids to treat irAEs. Therefore, cancer and ICIs immunotherapy should be considered as a possible basis for MTB susceptibility.

Although acute TB infection is diagnosed according to clinical symptoms, chest X-ray, and sputum culture or PCR, the initial symptoms and radiological findings may not distinguish between tumor progression, TB infection, or changes in immune-mediated imaging. Then, IGRA testing should be performed before the treatment of immune checkpoint inhibitors therapy to rule out the possibility of latent Mycobacterium TB infection. Only one of these patients took IGRA testing before ICIs administration. The IGRA result of the patient, which was negative at first, changed to positive after new abnormalities on imaging, which contributed to the diagnosis of TB. Therefore, it is necessary to take IGRA testing before the treatment of immune checkpoint inhibitors therapy.

It is an important clinical question whether ICIs therapy can be continued after the development of active TB. Given that continued treatment with ICIs may worsen TB, it is reasonable to discontinue ICIs after the diagnosis of active TB. However, the appropriate time to restart ICIs therapy is still being worked out. Although the four reported patients who restarted immunotherapy did not have a relapse or worsening of TB, these four cases are not sufficient to confirm the appropriateness of restarting ICIs therapy in the presence of TB. In clinical practice, the decision to continue, temporarily interrupt, or permanently suspend treatment with ICIs should take several aspects into account, including the severity of MTB infection, tumor control, and the severity of complications. Therefore, it is better to let multidisciplinary specialists in oncology discuss whether ICIs can be restarted or not.

Above all, ICIs have revolutionized cancer treatment, namely, greatly improving the overall survival of patients. However, potential MTB infection or reactivation of the disease during the treatment with ICIs still needs to be considered. It is recommended that IGRA testing be performed before ICIs therapy, and the development of TB during immunotherapy should be closely monitored for in patients who are positive in the results of IGRA testing to enable early diagnosis and timely treatment.

## Data availability statement

The original contributions presented in the study are included in the article/**Supplementary Materials** further inquiries can be directed to the corresponding author/s.

## Ethics statement

The studies involving human participants were reviewed and approved by Shengli Oilfield Central Hospital. The patients/participants provided their written informed consent to participate in this study. Written informed consent was obtained from the individual(s) for the publication of any potentially identifiable images or data included in this article. The authors are accountable for all aspects of the work in ensuring that questions related to the accuracy or integrity of any part of the work are appropriately investigated and resolved. The study was conducted in accordance with the Declaration of Helsinki (as revised in 2013). The study was approved by the Institutional Review Board of Shengli Oilfield Central Hospital (No. 2022026). All the study subjects provided informed consent.

## Author contributions

SL, CL, and GX designed the overall research strategy. CL collected the clinical information. CL and GX wrote the manuscript. CL, GX, TF, and SG participated in data discussion. All authors contributed to the article and approved the submitted version. CL and GX contributed equally to this work and share first authorship.
