# Supplementary material for: Tuberculosis infection following immune checkpoint inhibitor treatment for advanced cancer: a case report and literature review
Source: Front Immunol. 2023 May 25;14:1162190. doi: 10.3389/fimmu.2023.1162190 (PMC10248429; doi:10.3389/fimmu.2023.1162190)
Supplement: Supplementary file 1 [file Table_1.docx]

**Supplementary file**

**Table S1 Summary analysis of clinical and literature cases.**

| **Study objects** | **Age/Gender/Nationality** | **Cancer types** | **Immune checkpoint inhibitors (ICIs)** | **Time lag (months) from ICI initiation to TB diagnosis** | **Regimens before ICI therapy** | **Application of other immunosuppressants** | **Diagnosis of TB** | **Treatment of TB** | **Whether to discontinue ICI therapy** | **Whether to restart ICIs** | **Outcomes** |
| --- | --- | --- | --- | --- | --- | --- | --- | --- | --- | --- | --- |
| Lee 2016 (1) | 87/Male/Chinese | Hodgkin lymphoma | Pembrolizumab | 4.5 | ABVD, XRT | - | Sputum culture (+) | Lsoniazid, Rifampicin, Ethambutol | Yes | No | TB had been completely relieved. |
| Fujita 2016 (2) | 72/Male/Japanese | Lung squamous cell carcinoma | Nivolumab | 4 | Carboplatin + Docetaxel Carboplatin + Gemcitabine | - | BAL culture (+), PCR (+) | Unknown | Yes | No | Unknown |
| Chu 2017 (3) | 59/Male/Unknown | Lung adenocarcinoma | Nivolumab | 1.5 | Gefitinib | Prednisone | Pericardial fluid culture (+), (pericardial) Histology | Specific medication is not specified. | Yes | Yes | Pericardial fluid had completely subsided. |
| Jensen 2018 (4) | 56/Male/Caucasian | Lung adenocarcinoma | Nivolumab | 9 | Pemetrexed | - | Lung AFB (+), PCR (+), Histology | Specific medication is not specified. | Yes | No | Unknown |
| Picchi 2018 (1) (5) | 50/Male/Caucasian | Melanoma | Pembrolizumab | 3 | Unknown | - | TST (+), (pleural) Histology | The “four-drug” regimen | No | No | Hydrothorax had completely subsided. |
| Picchi 2018 (2) (5) | 64/Male/Caucasian | Non-small cell lung cancer | Nivolumab | 1 | Unknown | - | Bone culture (+), PCR (+), Histology | The “four-drug” regimen | Yes | No | Died after the surgery for spinal cord compression. |
| Tetikkurt 2018 (6) | 53/Male/Unknown | Oral squamous cell carcinoma | Pembrolizumab | Unknown | Surgical resection, Cisplatin + Radiotherapy | - | PCR (+) | Specific medication is not specified. | Yes | Yes | Tumour had shrunk after the reapplication of ICIs. |
| He 2018 (7) | 65/Female/Chinese | Melanoma | Pembrolizumab | 9.25 | IL-2 | - | Lung AFB (+), Histology, BAL culture (+), PCR (+) | HREZ, Streptomycin, ethambutol, moxifloxacin were administered due to hepatic damage, fever | Yes | Yes | Tumor had been completely relieved after the combination of ICI and anti-TB therapy. |
| Takata 2019 (8) | 75/Male/Japanese | Lung adenocarcinoma | Nivolumab | 10.5 | Carboplatin + Pemetrexed Carboplatin + Paclitaxelalbumin | - | Sputum culture (+), PCR (+) | HREZ, Isoniazid, rifampicin were administered due to abnormal reaction | Yes | Yes | TB did not recur after the reapplication of ICI. |
| Barber 2019 (1) (9) | 59/Male/Vietnamese | Nasopharyngeal carcinoma | Nivolumab | 1.5 | Unknown | - | Lung AFB (+), Histology, Sputum PCR (+) | HREZ, Streptomycin, rifampicin, moxifloxacin, linezolid were administered due to intestinal perforation | Yes | No | Died 2 months after the application of ICI because of the worsening of the primary disease. |
| Barber 2019 (2) (9) | 83/Male/Caucasian | Merkel cell carcinoma | Pembrolizumab | 8.25 | Unknown | - | Lung AFB (+), Histology | HREZ，Levofloxacin, rifapentin were administered after isoniazid administration | Yes | Yes | Tumor had shrunk after the reapplication of ICI because of disease progression after completion of anti-TB treatment. |
| Tsai 2019 (10) | 49/Male/Unknown | hard palate squamous cell carcinoma | Nivolumab | 3 | Cisplatin + Radiotherapy, Cetuximab, Paclitaxel, Carboplatin | - | Sputum PCR (+), PCR (+), Culture (+) | Specific medication is not specified. | Yes | No | Died of acute respiratory failure caused by bacterial pneumonia secondary to pulmonary TB. |
| van Eeden 2019 (11) | 56/Female/Caucasian | Lung adenocarcinoma | Nivolumab | Unknown | Gemcitabine + Carboplatin，Pemetrexed + Radiotherapy | - | Sputum PCR (+) | HREZ | Yes | Yes | The patient's TB improved, but died of disease progression after restarting with ICI. |
| Inthasot 2020 (12) | 69/Male/Belgian | Lung adenocarcinoma | Nivolumab | 13 | Cisplatin + Pemetrexed Pemetrexed maintenance | - | BAL PCR, Culture (+) | Specific medication is not specified. | Unknown | NO | Unknown |
| Elkington 2018 (13) | 62/Male/Unknown | Ocular melanoma | Pembrolizumab + Ipilimumab | 24 | Surgical resection | - | BAL culture (+), (liver and lung) Histology | Specific medication is not specified. | Unknown | NO | Clinical symptoms, liver function and lung lesions improved. |
| Kim 2018 (14) | 60/Male/Korean | Undifferentiated lung cancer | Nivolumab | 15 | Unknown | - | (Endobronchial lesions) PCR (+), Histology | Specific medication is not specified. | Stop for 1 month | Yes | TB had improved after medication, but liver function abnormalities occurred after the reapplication of ICI. Then the patient was treated with liver protective after discontinuing the administration of anti-TB and ICI. |
| Suliman2021 (15) | 58/Male/Caucasian | Lung adenocarcinoma | Pembrolizumab | 3 | - | - | BAL AFB (+), PCR (+) | HREZ | Yes | No | Being currently treated with anti-TB and chemotherapy and is clinically stable. |
| Anastasopoulou2019 (1) (16) | 76/Female/Greek | Melanoma | Nivolumab | 6 | INF | Methylprednisolone + Infliximab | BAL PCR, Culture (+) | HREZ | No | No | Died of acute respiratory failure 3 days after anti-TB therapy. |
| Anastasopoulou2019 (2) (16) | 85/Male/Greek | Melanoma | Atezolizumab | 6.75 | INF | - | Sputum culture (+) | Isoniazid, rifampicin, pyrazinamide | Yes | NO | Pulmonary TB had been completely relieved. |
| The patient | 68/Male/Chinese | Lung squamous cell carcinoma | Camrelizumab | 1 | Docetaxel + Cisplatin | - | BAL AFB (+) | HREZ | Yes | NO | Being currently treated with anti-TB and chemotherapy and is clinically stable. |

**Abbreviations:** BAL: bronchoalveolar lavage fluid; TST: tuberculin skin test; AFB: acid fast bacilli; PCR: polymerase chain reaction; hrze: isoniazid, rifampicin, pyrazinamide, ethambutol.

Note: In the column of Immune checkpoint inhibitors (ICIs): Nivolumab, Pembrolizumab and Camrelizumab are anti-PD-1 ICIs, Atezolizumab is anti-PD-L1 ICI, Ipilimumab is anti-CTLA-4 ICI.

**Reference**

1. Lee JJX, Chan A, Tang T. Tuberculosis reactivation in a patient receiving anti-programmed death-1 (PD-1) inhibitor for relapsed Hodgkin’s lymphoma. *Acta Oncol* (2016) 55:519–520. doi: 10.3109/0284186X.2015.1125017

2. Fujita K, Terashima T, Mio T. Anti-PD1 Antibody Treatment and the Development of Acute Pulmonary Tuberculosis. *J Thorac Oncol* (2016) 11:2238–2240. doi: 10.1016/j.jtho.2016.07.006

3. Chu Y-C, Fang K-C, Chen H-C, Yeh Y-C, Tseng C-E, Chou T-Y, Lai C-L. Pericardial Tamponade Caused by a Hypersensitivity Response to Tuberculosis Reactivation after Anti–PD-1 Treatment in a Patient with Advanced Pulmonary Adenocarcinoma. *Journal of Thoracic Oncology* (2017) 12:e111–e114. doi: 10.1016/j.jtho.2017.03.012

4. Jensen KH, Persson G, Bondgaard A-L, Pøhl M. Development of pulmonary tuberculosis following treatment with anti-PD-1 for non-small cell lung cancer. *Acta Oncol* (2018) 57:1127–1128. doi: 10.1080/0284186X.2018.1433877

5. Picchi H, Mateus C, Chouaid C, Besse B, Marabelle A, Michot JM, Champiat S, Voisin AL, Lambotte O. Infectious complications associated with the use of immune checkpoint inhibitors in oncology: reactivation of tuberculosis after anti PD-1 treatment. *Clin Microbiol Infec* (2018) 24:216–218. doi: 10.1016/j.cmi.2017.12.003

6. Tetikkurt S, Taş F, Emre F, Özsoy Ş, Bilece ZT. Significant Neutrophilic Emperipolesis in Squamous Cell Carcinoma. *Case Rep in Oncol Med* (2018) 2018:1–5. doi: 10.1155/2018/1301562

7. He W, Zhang X, Li W, Kong C, Wang Y, Zhu L, Xu R, Deng G, Zhang P. Activated pulmonary tuberculosis in a patient with melanoma during PD-1 inhibition: a case report. *OTT* (2018) Volume 11:7423–7427. doi: 10.2147/OTT.S178246

8. Takata S, Koh G, Han Y, Yoshida H, Shiroyama T, Takada H, Masuhiro K, Nasu S, Morita S, Tanaka A, et al. Paradoxical response in a patient with non-small cell lung cancer who received nivolumab followed by anti-Mycobacterium tuberculosis agents. *J Infect Chemother* (2019) 25:54–58. doi: 10.1016/j.jiac.2018.06.016

9. Barber DL, Sakai S, Kudchadkar RR, Fling SP, Day TA, Vergara JA, Ashkin D, Cheng JH, Lundgren LM, Raabe VN, et al. Tuberculosis following PD-1 blockade for cancer immunotherapy. *Sci Transl Med* (2019) 11:eaat2702. doi: 10.1126/scitranslmed.aat2702

10. Tsai C-C, Chen J-H, Wang Y-C, Chang F-Y. Re-activation of pulmonary tuberculosis during anti-programmed death-1 (PD-1) treatment. *QJM: Int J Med* (2019) 112:41–42. doi: 10.1093/qjmed/hcy243

11. van Eeden R, Rapoport BL, Smit T, Anderson R. Tuberculosis Infection in a Patient Treated With Nivolumab for Non-small Cell Lung Cancer: Case Report and Literature Review. *Front Oncol* (2019) 9:659. doi: 10.3389/fonc.2019.00659

12. Inthasot V, Bruyneel M, Muylle I, Ninane V. Severe pulmonary infections complicating nivolumab treatment for lung cancer: a report of two cases. *Acta Clin Belg* (2020) 75:308–310. doi: 10.1080/17843286.2019.1629078

13. Elkington PT, Bateman AC, Thomas GJ, Ottensmeier CH. Implications of Tuberculosis Reactivation after Immune Checkpoint Inhibition. *Am J Respir Crit Care Med* (2018) 198:1451–1453. doi: 10.1164/rccm.201807-1250LE

14. Kim TH, Kim J. P3.CR-16 A Case of Toxic Hepatic Event Occurring in Combination Treatment with Nivolumab and Anti-Tuberculosis in Advanced Lung Cancer. *J Thorac Oncol* (2018) 13:S1034. doi: 10.1016/j.jtho.2018.08.1995

15. Suliman AM, Bek SA, Elkhatim MS, Husain AA, Mismar AY, Eldean MZS, Lengyel Z, Elazzazy S, Rasul KI, Omar NE. Tuberculosis following programmed cell death receptor-1 (PD-1) inhibitor in a patient with non-small cell lung cancer. Case report and literature review. *Cancer Immunol Immun* (2021) 70:935–944. doi: 10.1007/s00262-020-02726-1

16. Anastasopoulou A, Ziogas DC, Samarkos M, Kirkwood JM, Gogas H. Reactivation of tuberculosis in cancer patients following administration of immune checkpoint inhibitors: current evidence and clinical practice recommendations. *J Immunother Cancer* (2019) 7:239. doi: 10.1186/s40425-019-0717-7
